# Sodium butyrate inhibits osteogenesis in human periodontal ligament stem cells by suppressing smad1 expression

**DOI:** 10.1186/s12903-022-02255-6

**Published:** 2022-07-19

**Authors:** Jingyi Hou, Junji Xu, Yi Liu, Haiping Zhang, Sihan Wang, Yao Jiao, Lijia Guo, Song Li

**Affiliations:** 1 Department of Orthodontics, School of Stomatology, Capital Medical University, Tian Tan Xi Li No.4, Beijing, 100050 People’s Republic of China; 2 Laboratory of Tissue Regeneration and Immunology and Department of Periodontics, Beijing Key Laboratory of Tooth Regeneration and Function Reconstruction, School of Stomatology, Capital Medical University, Beijing, People’s Republic of China; 3 Immunology Research Center for Oral and Systemic Health, Beijing Friendship Hospital, Capital Medical University, Beijing, People’s Republic of China

**Keywords:** Sodium butyrate, Periodontal ligament stem cells, Proliferation, Osteogenic differentiation, Smad1, Free fatty acids receptor 2

## Abstract

**Background:**

Butyrate is a major subgingival microbial metabolite that is closely related to periodontal disease. It affects the proliferation and differentiation of mesenchymal stem cells. However, the mechanisms by which butyrate affects the osteogenic differentiation of periodontal ligament stem cells (PDLSCs) remain unclear. Here, we investigated the effect of sodium butyrate (NaB) on the osteogenic differentiation of human PDLSCs.

**Methods:**

PDLSCs were isolated from human periodontal ligaments and treated with various concentrations of NaB in vitro. The cell counting kit-8 assay and flow cytometric analysis were used to assess cell viability. The osteogenic differentiation capabilities of PDLSCs were evaluated using the alkaline phosphatase activity assay, alizarin red staining, RT-PCR, western blotting and in vivo transplantation.

**Results:**

NaB decreased PDLSC proliferation and induced apoptosis in a dose- and time-depend manner. Additionally, 1 mM NaB reduced alkaline phosphatase activity, mineralization ability, and the expression of osteogenic differentiation-related genes and proteins. Treatment with a free fatty acids receptor 2 (FFAR2) antagonist and agonist indicated that NaB inhibited the osteogenic differentiation capacity of PDLSCs by affecting the expression of Smad1.

**Conclusion:**

Our findings suggest that NaB inhibits the osteogenic differentiation of PDLSCs by activating FFAR2 and decreasing the expression of Smad1.

**Supplementary Information:**

The online version contains supplementary material available at 10.1186/s12903-022-02255-6.

## Background

Periodontitis is a microbially associated chronic inflammatory disease that is characterized by loss of periodontal tissue integrity [1]. In the Global Burden of Disease 2010 study, severe periodontitis was reported as the sixth most prevalent health condition worldwide [2]. During the inflammation period of periodontitis, bacterial cytotoxins and metabolites can promote the host defence response, which increases the secretion of protein-rich gingival crevicular fluid and facilitates the growth of pathogenic periodontal bacteria [3, 4].

Short chain fatty acids (SCFAs), including butyrate, are the major metabolic by-products produced by subgingival pathogenic bacteria, such as *Porphyromonas gingivalis* and *Fusobacterium nucleatum* [4–6]. Higher concentration of butyrate can be detected in the gingival crevicular fluid of patients with periodontitis [7, 8]. In the oral cavity, butyrate decreases cytokine-induced intercellular adhesion molecule-1 expression in epithelial cells, and destroys gingival epithelial cell homeostasis by reducing the intercellular junctions [9, 10]. In addition, butyrate causes gingival epithelial cell autophagy via the AMP-activated protein kinase signalling pathway and results in subsequent cell death [11]. Butyrate exposure promotes gingival fibroblast apoptosis via extrinsic and intrinsic pathways and increases pro-inflammatory cytokine production [12].

Mesenchymal stem cells (MSCs) have self-renewal and multi-lineage differentiation abilities, and play major roles in periodontal tissue regeneration [13]. Recent studies have shown that 0.5 μM sodium butyrate (NaB) promotes the osteogenic and dentinogenic differentiation of mouse dental pulp stem cells by inhibiting histone deacetylation [14]. However, 0.5 mM NaB decreases mineralised nodule formation and the expression of runt-related transcription factor 2 (*Runx2*), osteocalcin (*OCN*), and type I collagen (*Col I*) in mouse bone marrow MSCs [15]. Periodontal ligament stem cells (PDLSCs) possess multiple differentiation capabilities, including differentiation into osteogenic and adipogenic cells [16]. Human PDLSCs have potential for use in cell-based treatments for mediating periodontal regeneration [17]. However, the effect of butyrate on PDLSCs remains unclear.

Butyrate can act as a signalling molecule that binds to free fatty acid receptors (FFAR), which are expressed in various tissues [18–20]. FFAR2 plays a major role in orthodontic tooth movement in C57BL/6 mice, and adipogenic differentiation of human adipose-derived MSCs [21, 22]. Smad proteins function as major signalling mediators during the osteogenic differentiation of MSCs. When receptor-associated Smad1/5/8 is phosphorylated by the activin receptor-like kinase 1, it forms a complex with Smad4 and translocates into the nucleus, where it regulates the expression of osteogenic genes, such as *Runx2* and osterix (*OSX)* [23–25]. Here, we investigate the effects of NaB on human PDLSC osteogenesis and the signalling pathways that influence the biological behaviour of PDLSCs.

## Methods

### Cell culture of human PDLSCs

All experiments were performed according to the ISSCR Guidelines for Stem Cell Research and Clinical Translation, and approved by the Ethics Committee of Capital Medical University School of Stomatology (Beijing, China) (CMUSH-IRB-KJ-PJ-2020-11). Human premolars and impacted third molars were collected from healthy patients at the Department of Oral and Maxillofacial Surgery in Affiliated Stomatological Hospital of Capital Medical University. Informed consents were obtained from all donors. PDLSCs were isolated and cultured according to previously reported protocols [16]. The periodontal ligament tissues were gently separated from the middle third of the tooth roots and digested with 3 mg/ml collagenase type I (Sigma-Aldrich, USA) and 4 mg/ml dispase (Sigma-Aldrich, USA) for 1 h at 37 °C. After digestion, the tissues were cultured in α-modified Eagle's medium (Gibco, USA) containing 20% fetal bovine serum (Gibco, USA), 1% penicillin/streptomycin (Gibco, USA) and 1% glutamine (Gibco, USA) in 5% CO_2_ at 37 °C. PDLSCs from passages 3–5 were used in the following experiments.

### Immunofluorescence staining

PDLSCs were cultured on 24-well plates with glass coverslips at a density of 10^5^ cells/well overnight. Then, PDLSCs were fixed with 4% paraformaldehyde and incubated with anti-CD146 (1:100; Abcam, USA), anti-CD105 (1:100; Abcam, USA), and anti-CD45 (1:200; Abcam, USA) primary antibodies. The samples were then treated with rhodamine/FITC-conjugated secondary antibodies (1:1000; Sigma-Aldrich, USA) and stained with 4,6-diamidino-2-phenylindole (Sigma-Aldrich, USA). Jurkat cells were stained with anti-CD45 antibody as a positive control. The images were captured with a fluorescence microscopy (OLYMPUS, Japan).

### Flow cytometric analysis

For stem cell identification, 10^6^ PDLSCs were collected and fixed with 70% ethanol. Antibodies, including PE-labeled anti-CD146 (Biolegend, USA), FITC-labeled anti-CD105 (Biolegend, USA) and APC-labeled anti-CD45 (Biolegend, USA), were used to incubate the cells for 30 min in darkness. Jurkat cells were stained with anti-CD45 antibody as a positive control. Flow cytometry (FACS Calibur, BD Bioscience, USA) was used to test the samples.

PDLSCs were cultured on 6-well plates at a density of 10^6^ cells/well overnight and treated with 0, 1 and 5 mM NaB for 24 and 72 h. The cells were stained according to the manufacturer’s protocol (Annexin V-FITC Apoptosis Detection Kit, BD Bioscience, USA). The percentage of apoptosis rate was then detected by flow cytometry.

### Cell counting kit-8 (CCK-8) assay

Cell proliferation was measured using the Cell Counting Kit-8 (Dojindo, Japan) according to the manufacturer’s instruction. PDLSCs were cultivated on 96-well plates at a density of 5 × 10^3^ cells/well and treated with 0, 0.625, 1.25, 2.5, 5 mM NaB for 24, 48 and 72 h. The absorbance (OD) value was read at a wavelength of 450 nm.

### Alkaline phosphatase (ALP) activity assay and alizarin red staining

PDLSCs were cultivated on 6-well plates at a density of 2 × 10^5^ cells/well. When reaching 80–90% confluence, the culture medium was replaced by osteogenesis-inducing media containing 100 mM ascorbic acid, 2 mM β-glycerophosphate, and 10 nM dexamethasone. The cells were treated with 0, 0.1 and 1 mM NaB at the same time. Based on the preliminary data, we used 10 μM FFAR2-selective antagonist (GLPG0974, Sigma-Aldrich, USA) and 10 μM agonist (4-CMTB, MedChemExpresss, USA) to repress and activate FFAR2, respectively during PDLSC osteogenesis (Additional files 1, 2: Figs. S1, S2).

After 5 days of osteogenic induction, PDLSCs were stained with ALP capacity kit (Sigma-Aldrich, USA). The OD value was read at a wavelength of 495 nm. The results were standardized on the basis of protein concentration. After 2 weeks of induction, PDLSCs were fixed with 70% ethanol for 1 h, and 1% alizarin red solution was utilized to stain the mineralized nodules (Solarbio, China). The relative concentration of calcium was measured after solubilizing in 10% cetylpyridinium chloride (Sigma-Aldrich, USA) for 30 min. The OD value was read at a wavelength of 562 nm.

### Adipogenic differentiation assay

PDLSCs were cultivated on 6-well plates at a density of 2 × 10^5^ cells/well. When reaching 80–90% confluence, the culture medium was replaced by adipogenesis-inducing medium (Cyagen Biosciences Inc, China). After 21 days of adipogenic induction, PDLSCs were fixed with 4% paraformaldehyde for 30 min. Cells were stained with Oil red O solution at room temperature for 15 min. The images were captured with an inverted microscopy (OLYMPUS, Japan).

### Real-time reverse transcriptase-polymerase chain reaction (real-time RT-PCR)

Following 7 and 14 days of induction, total RNA was isolated from PDLSCs by using Trizol reagents (Cwbio, China). Reverse transcription was completed according to the manufacturer’s protocol (Takara, China). Then, RT-PCR reactions were performed using the SYBR Premix Ex Taq™ (Takara, China) and an icycler iQ Multi-color Real-time PCR Detection System. The primers for specific genes are listed in Table 1.

**Table 1:** Primers sequences used in the real-time RT-PCR

Gene symbol	Primer sequences (5′-3′)
*GAPDH-F*	CGGACCAATACGACCAAATCCG
*GAPDH-R*	AGCCACATCGCTCAGACACC
*RunX2-F*	GACTGTGGTTACCGTCATGGC
*RunX2-R*	ACTTGGTTTTTCATAACAGCGGA
*OSX-F*	CCTCCTCAGCTCACCTTCTC
*OSX-R*	GTTGGGAGCCCAAATAGAAA
*OCN-F*	CAGACAAGTCCCACACAGCA
*OCN-R*	CTTGGCATCTGTGAGGTCAG
*OPN-F*	AGCCACATCGCTCAGACACC
*OPN-R*	TGAAATTCATGGCTGTGGAA

### Western blotting analysis

Following 7 and 14 days of osteogenic induction, total protein was obtained from PDLSCs. The protocol has been described previously.^14^ The following primary antibodies were used: anti-Runx2 (1:1000; Cell Signaling Technology, USA), anti-Smad1 (1:1000; Cell Signaling Technology, USA), anti-p-Smad1/5/8 (1:1000; Cell Signaling Technology, USA), anti-OCN (1:1000; Abcam, USA), anti-osteopontin (anti-OPN) (1:1000; Abcam, USA), and anti-OSX (1:1000; Abcam, USA). GAPDH (1:2000; Abclonal, China) was used as a control.

### Transplantation in nude mice

Six 10-week-old immunocompromised beige mice (nu/nu nude mice) were purchased from the Institute of Animal Science of the Vital River (Beijing, China). The experiments were performed according to ARRIVE guidelines, and institutionally set guidelines for animal research approved by the Animal Care and Use Committee of the Beijing Stomatological Hospital, Capital Medical University, Beijing, China (KQYY-201907–003). Approximately 2 × 10^6^ human PDLSCs treated with or without 1 mM NaB were mixed with 20 mg hydroxyapatite/tricalcium phosphate (HA/TCP) ceramic particles (Engineering Research Center for Biomaterials, Sichuan University, China). Then, the mixtures were transplanted separately into the dorsal surface of the mice. After 3 months, the implants were harvested, fixed with 4% paraformaldehyde, and decalcified with 10% EDTA in PBS (pH 7.4) at 4 °C. The decalcified samples were then dehydrated and embedded in paraffin. The samples were sectioned at 5 μm and stained with hematoxylin and eosin (H&E). Six sections were evaluated per sample, and the blind evaluation was performed. The bone-like areas were measured using the Image-Pro Plus 6.0 program.

### Statistical analysis

Statistical analysis was performed using SPSS 26.0 software. The Student’s t test or one-way ANOVA was used to assess statistical significance, with a *p* ≤ 0.05 was regarded as significant.

## Results

### Human PDLSCs culture and identification

**Fig. 1:**
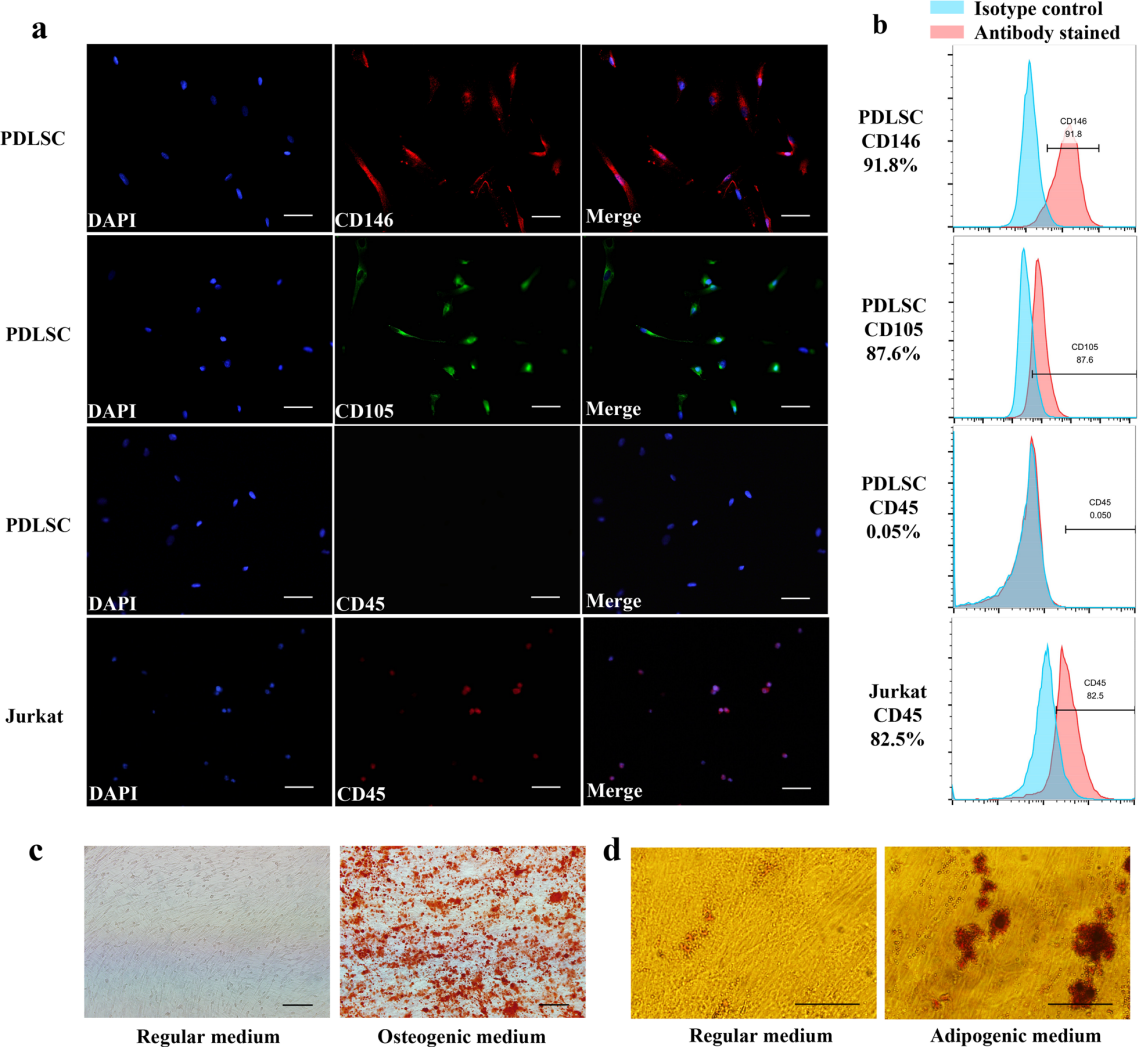
Identification of human periodontal ligament stem cells (PDLSCs). **a** Immunofluorescence showed that PDLSCs used in this study express CD146 and CD105, but negative for CD45. Jurkat cells were positive for CD45. Scale bars, 100 μM. **b** Flow cytometry showed that human PDLSCs exhibited the expression of cell surface markers, including CD146 (91.8%), CD105 (87.6%) and CD45 (0.05%). Isotype control was used in this experiment. Jurkat cells exhibited the expression of CD45 (82.5%). **c** Alizarin red staining assay was performed to prove the mineralized nodules formation after 14-days osteogenic induction. Scale bars, 100 μm. **d** The cellular Oil red O-positive lipid clusters were stained after 21-days adipogenic induction. Scale bars, 50 μm

Human PDLSCs displayed a long spindle shape after passage. Flow cytometry and immunofluorescence revealed that human PDLSCs expressed MSC markers, including CD146 and CD105. Different from Jurkat cells, PDLSCs were negative for CD45 (Fig. 1a, b). The formation of calcium deposits and Oil red O-positive lipid clusters proved that PDLSCs had osteogenic and adipogenic differentiation abilities (Fig. 1c, d). These results demonstrated that human PDLSCs had characteristics similar to those of MSCs.

### Effects of NaB on the proliferation and apoptosis of PDLSCs

**Fig. 2:**
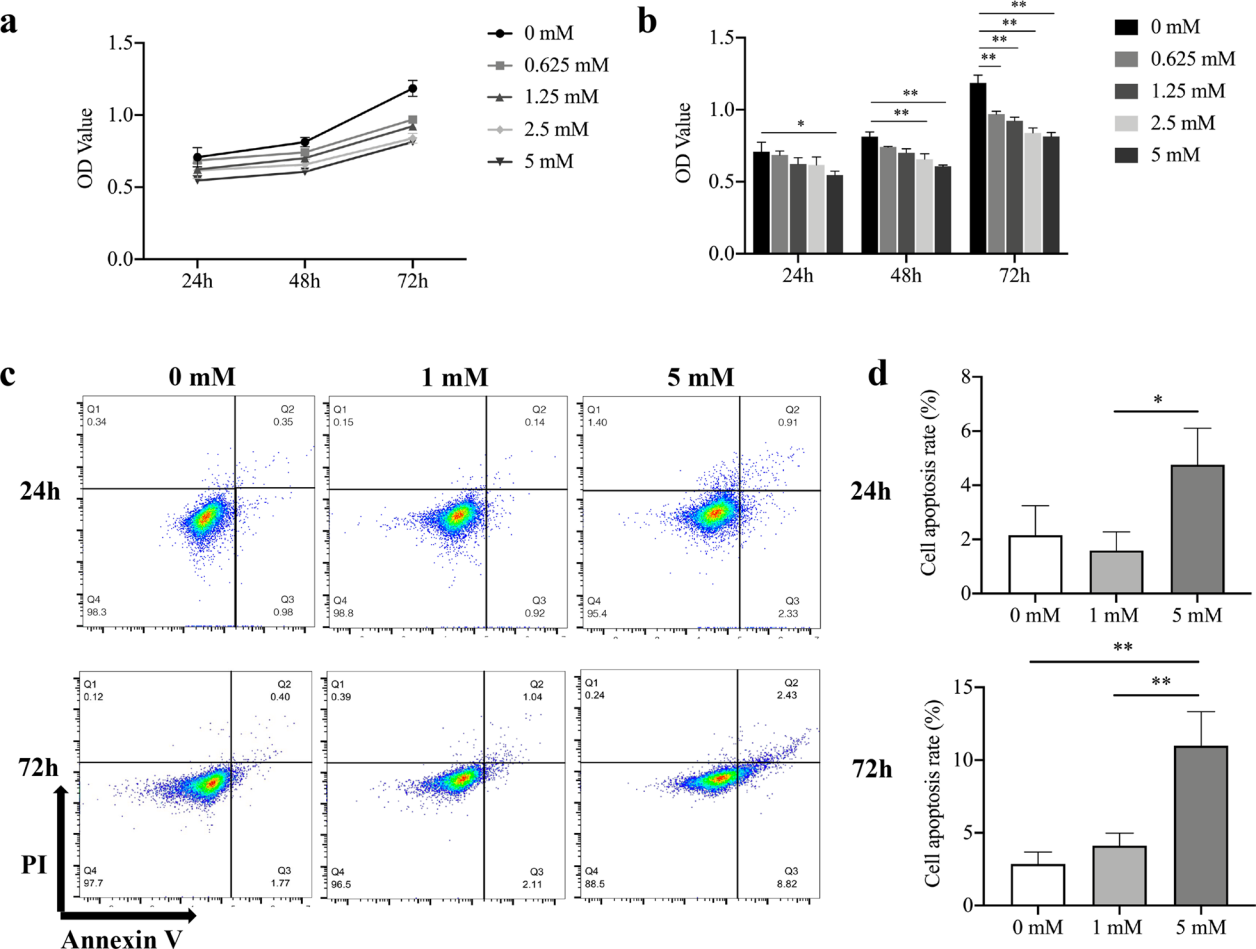
Sodium butyrate (NaB) reduced the viability of periodontal ligament stem cells (PDLSCs). **a,** The cell proliferation curve of PDLSCs was depicted after the treatment of NaB for 24, 48, and 72 h. **b,** The absorbance values showed that NaB inhibited PDLSC proliferation in a dose- and time-dependent manner. **c, d** Flow cytometry showed that 5 mM NaB induced human PDLSC apoptosis in 24 and 72 h. Error bars represent the standard deviation (n = 3). *p < 0.05, **p < 0.01, using Student’s t test and one-way ANOVA

To explore the effects of NaB on cell proliferation, PDLSCs were treated with different concentrations of NaB. The results indicated that the cell proliferation decreased in a dose- and time-dependent manner (Fig. 2a, b). The cell proliferation was significantly decreased by 5 mM NaB after 24 h of treatment. After 48 h of treatment, the cell proliferation was markedly inhibited by 2.5 mM NaB and 5 mM NaB. A significant decrease was found in each concentration of NaB after 72 h of treatment. Additionally, flow cytometry demonstrated that 5 mM NaB significantly induced higher apoptosis rates at 24 and 72 h, while 1 mM NaB did not have a significant effect on PDLSC apoptosis (Fig. 2c, d).

### NaB inhibited the osteogenic differentiation of PDLSCs

**Fig. 3:**
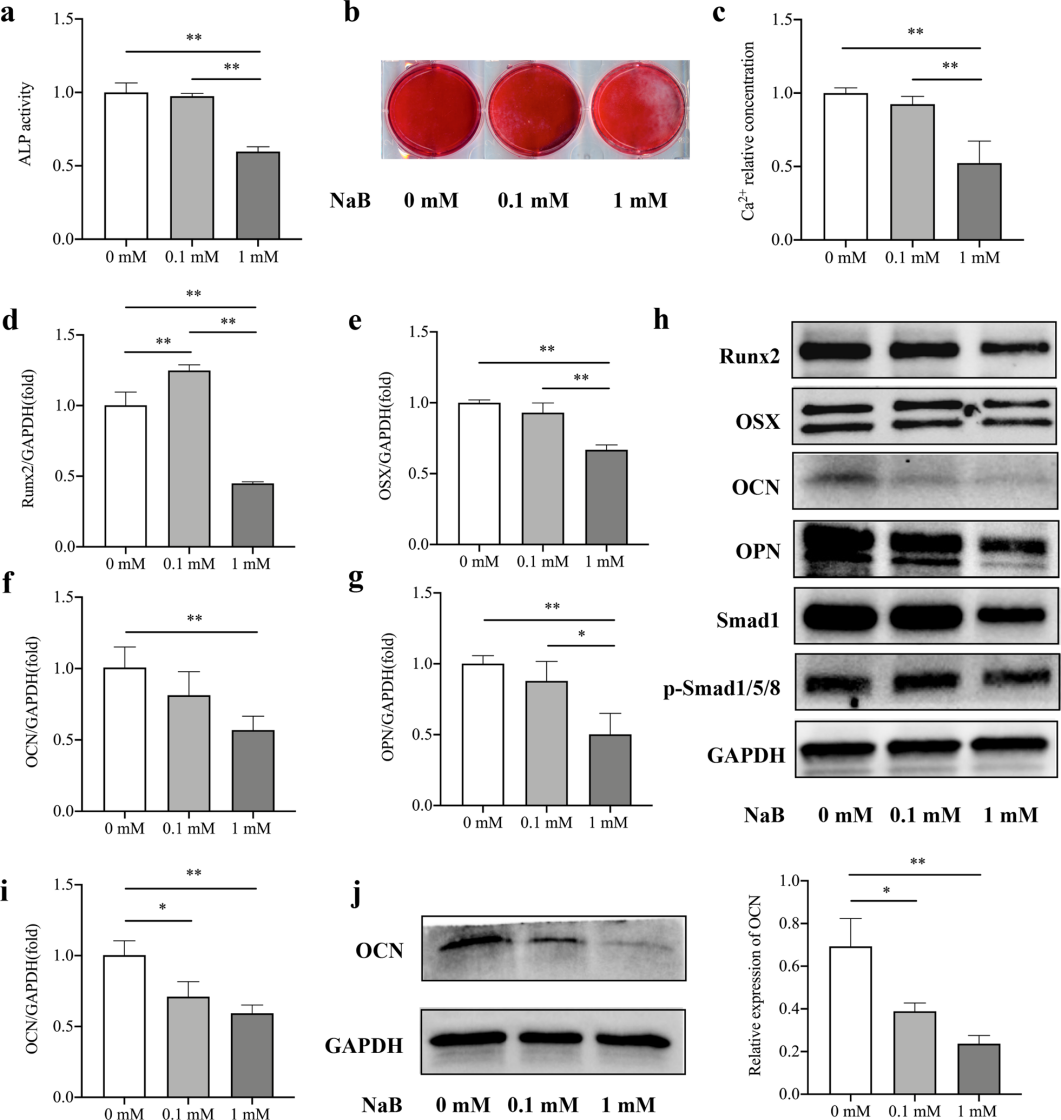
Sodium butyrate (NaB) decreased the osteogenic capacity of periodontal ligament stem cells (PDLSCs). **a** Evaluation of alkaline phosphatase (ALP) activity 5 days after osteogenic induction indicated that 1 mM NaB decreased ALP activity in PDLSCs. **b, c** Alizarin red staining assay and cetylpyridinium chloride assay performed 14 days after induction showed that 1 mM NaB decreased the formation of mineralized nodules in PDLSCs. **d–g** Following 7 days of osteogenic induction, RT-PCR showed that 1 mM NaB downregulated *Runx2*, *OSX*, *OCN*, and *OPN*. **h** Western blotting showed that 1 mM NaB reduced the protein expression of Runx2, OSX, OCN, OPN, Smad1 and p-Smad1/5/8. **i** Following 14 days of osteogenic induction, RT-PCR showed that 0.1 mM and 1 mM NaB downregulated *OCN*. **j** Western blotting and densitometric analysis showed that 0.1 mM and 1 mM NaB reduced the protein expression of OCN. The length of the blots was appropriate in the manuscript. Error bars represent the standard deviation (n = 3). *p < 0.05, **p < 0.01, using one-way ANOVA

PDLSCs were cultured in osteogenesis-inducing media. After 5 days of osteogenesis induction, 1 mM NaB decreased ALP activity by 50–60% (compared to 0 mM and 0.1 mM NaB) (Fig. 3a). After 2 weeks of induction, the formation of calcium deposits decreased in the 1 mM NaB group (compared to the 0 mM and 0.1 mM groups) (Fig. 3b, c). The effects of NaB on the osteogenic differentiation of PDLSCs were also evaluated by RT-PCR and western blotting. The results showed that after 7 days of induction, the expression of Runx2, OSX, OCN, OPN, Smad1 and p-Smad1/5/8 in the 1 mM NaB group was markedly lower than that in the 0 mM and 0.1 mM groups (Fig. 3d–h). After 14 days of induction, the expression of OCN in the 0 mM and 0.1 mM groups was significantly decreased compared to the 0 mM group (Fig. 3i, j).

### NaB downregulated Smad1 by activating FFAR2 in PDLSCs

**Fig. 4:**
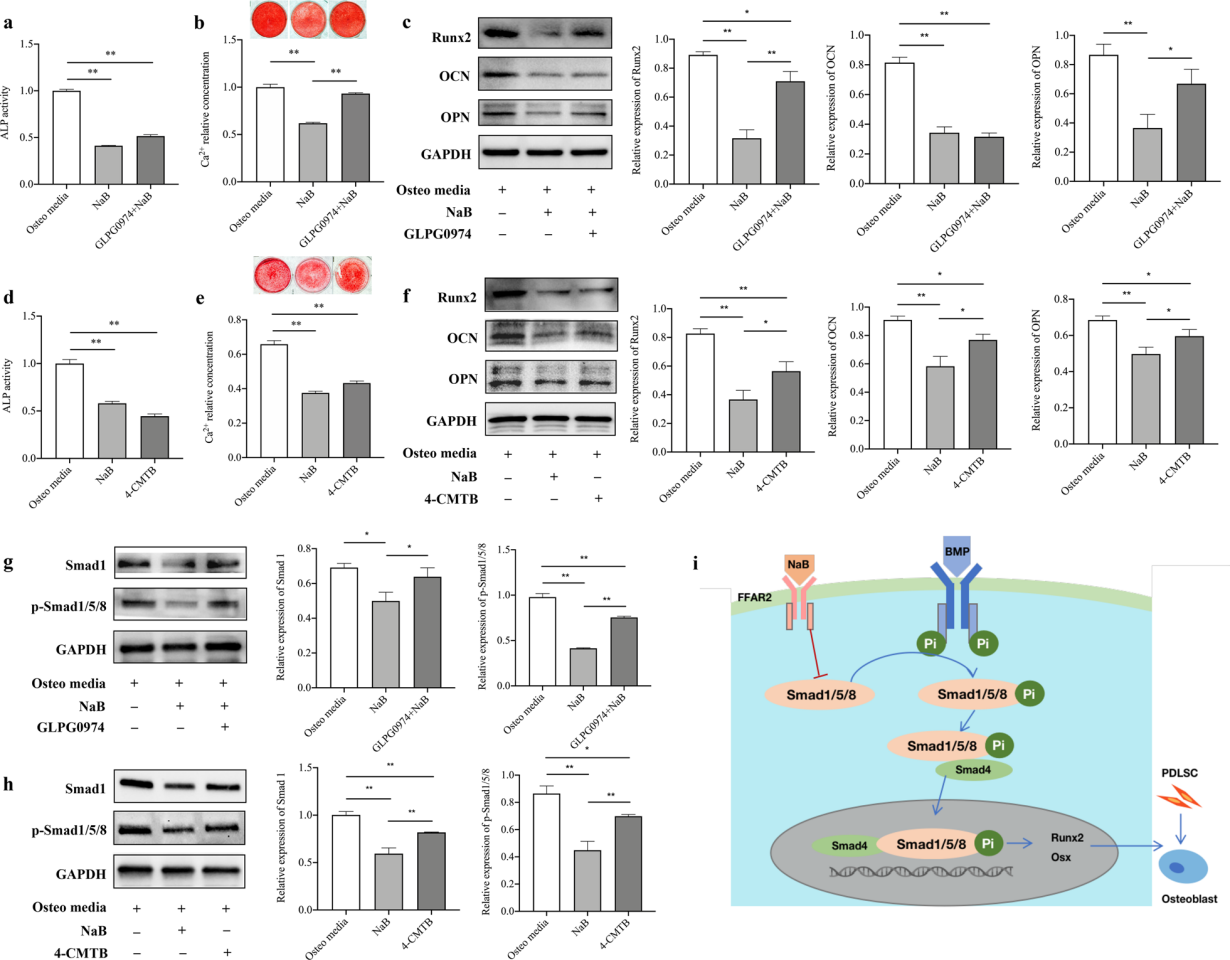
Sodium butyrate (NaB) inhibited periodontal ligament stem cell (PDLSC) osteogenesis by downregulating Smad1. **a** Free fatty acid receptor 2 (FFAR2) antagonist (GLPG0974) slightly increased alkaline phosphatase (ALP) activity in NaB-treated cells (compared to the NaB group). **b, c** GLPG0974 significantly reversed the NaB-mediated inhibition of mineralized nodule formation and the expression of Runx2 and OPN. **d–f** FFAR2 agonist (4-CMTB) decreased ALP activity, mineralized nodule formation and the expression of Runx2, OCN and OPN in NaB-treated cells. **g, h** Western blotting and densitometric analysis showed that GLPG0974 reversed the NaB-mediated reduction of Smad1 and p-Smad1/5/8 (compared to the NaB group), while 4-CMTB reduced the the expression of Smad1 and p-Smad1/5/8. The length of the blots was appropriate in the manuscript. **i** NaB decreased the expression of Smad1 by activating FFAR2, thus reducing the translocation of the p-Smad1/5/8-Smad4 complex into the nucleus, downregulating the expression of Runx2 and OSX, and inhibiting the osteogenic differentiation of PDLSCs. Error bars represent the standard deviation (n = 3). *p < 0.05, **p < 0.01, using one-way ANOVA

GLPG0974 treatment in the background of NaB stimulation slightly increased ALP activity (compared to that in the NaB group), but this was still lower than that detected in the control group (Fig. 4a). GLPG0974 significantly reversed the effect of NaB with respect to mineralised nodule formation, and expression of Runx2 and OPN (but not OCN) (Fig. 4b, c). Similar to that in the NaB group, 4-CMTB decreased ALP activity, mineralised nodule formation, and the expression of Runx2, OCN, and OPN (Fig. 4d–f). Western blotting showed that GLPG0974 treatment in the background of NaB stimulation reversed the reduction in Smad1 and p-Smad1/5/8 expression (compared to the NaB group), and the expression of Smad1 and p-Smad1/5/8 was reduced in response to 4-CMTB treatment (Fig. 4g, h). Figure 4i shows the mechanism proposed to explain these results, whereby NaB decreases the expression of Smad1 by activating FFAR2, thereby reducing p-Smad1/5/8-Smad4 translocation into the nucleus and downregulating Runx2 and OSX. This ultimately results in the inhibition of PDLSC osteogenic differentiation.

### NaB decreased human PDLSC-mediated bone formation in nude mice

**Fig. 5:**
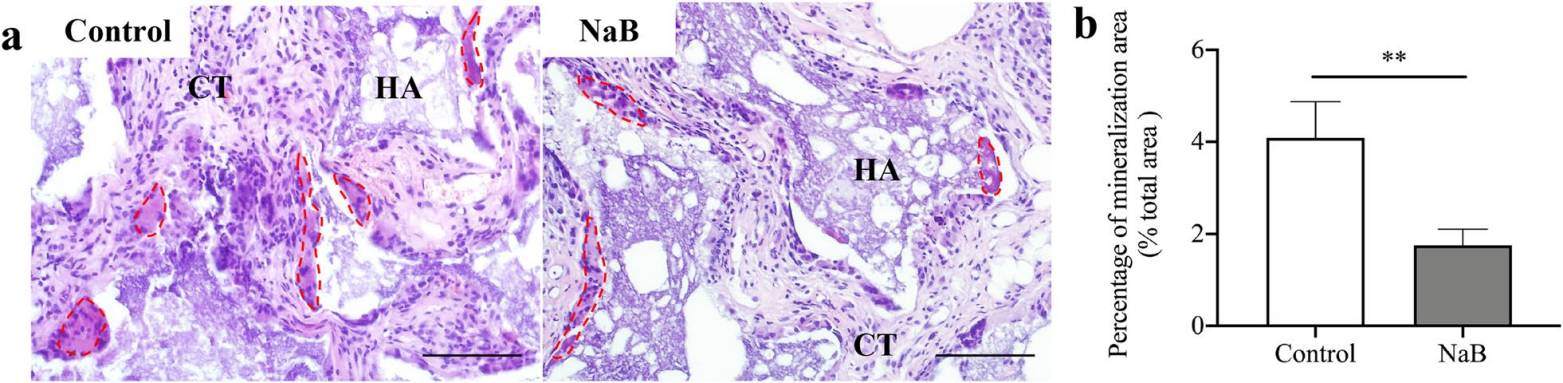
Sodium butyrate (NaB) decreased human periodontal ligament stem cell-mediated bone formation in nude mice. **a** H&E staining revealed less bone-like tissue formation around HA/TCP in the NaB-treated group compared to the control. **b** Quantitative measurement of the mineralization area revealed less bone-like tissue formation in the NaB-treated group than in the control. Red lines represent bone-like tissue; HA = HA/TCP; CT = connective tissue. Scale bars, 100 μM. Error bars represent the standard deviation (n = 3). *p < 0.05, **p < 0.01, using Student’s t test

To explore the effect of NaB on the osteogenic differentiation of PDLSCs in vivo, human PDLSCs were treated with or without NaB and transplanted into the dorsal surfaces of nude mice. NaB-treated PDLSCs generated less bone-like mineralised tissue than untreated PDLSCs (Fig. 5a). Quantitative measurement of the mineralisation area showed less bone-like tissue formation in the NaB-treated group than in the control group (Fig. 5b).

## Discussion

In this study, we demonstrated that NaB decreased PDLSC proliferation and induced apoptosis in a dose- and time-depend manner. Additionally, 1 mM NaB reduced the osteogenesis ability in PDLSC. Treatment with a FFAR2 antagonist and agonist indicated that NaB inhibited the osteogenic differentiation capacity of PDLSCs by affecting the expression of Smad1.

Gut and oral bacteria are the major metabolic sources of SCFAs. SCFAs produced in the gut can be passively absorbed or actively transported by epithelial cells. Although butyrate is present at high concentrations in the colon, it does not inhibit the proliferation of normal epithelial cells because it is used as the main energy source for colonocytes. In contrast, because of the different metabolic patterns, butyrate accumulates three-fold in cancerous epithelial cells and affects cell growth as a histone deacetylase (HDAC) inhibitor [18]. A study proposed that the specific structure and metabolism of the crypt protected stem/progenitor cells that are located near the bottom of the crypt from butyrate-induced growth impairment [26]. In conclusion, the effect of butyrate is not only concentration dependent, but is also related to tissue structure, transportation, and metabolic patterns.

In the periodontal tissue, the gingival epithelium comprises squamous epithelial cells and has a thinner mucosal layer than the gut mucosa, which could allow butyrate to easily penetrate and remain within the gingiva [27]. A study has shown that butyrate downregulates intercellular junction‒related genes in human oral squamous cells [10]. It is speculated that unlike in the gut, lower concentrations of butyrate could penetrate the periodontal epithelial structure and impair the functions of human PDLSCs. Here, we verified that NaB inhibited human PDLSC proliferation and induced apoptosis in a dose- and time-dependent manner. The cell proliferation results indicated that 2.5 mM NaB reduced PDLSCs proliferation at 48 h and 72 h, and 5 mM NaB reduced cell proliferation at all time points. The cell apoptosis results demonstrated that 5 mM NaB induced PDLSCs apoptosis at 24 h and 72 h, while 1 mM NaB did not affect PDLSCs apoptosis. Thus, we used 1 mM NaB as the maximum concentration for cell osteogenic differentiation. We used 0.1 mM NaB to explore whether lower concentration of NaB inducing the osteogenic differentiation in PDLSCs.

To date, the effect of butyrate on osteogenesis remains unclear. Tomoko et al. demonstrated that NaB (< 1 mM) could induce mineralised nodule formation in human osteoblasts [28]. Butyrate-fed C57BL/6 J mice exhibited significantly higher tibial bone mass than the control group and a reduction in osteoclast number, while osteoblast number and OCN expression remained unchanged [29]. In contrast, Chang et al. found that NaB decreased Col I expression in MG-63 osteoblasts (between 43 and 77% at concentrations ranging between 2 and 16 mM, respectively) [30]. Here, 0.1 mM NaB only decreased the expression of OCN after 14 days osteogenic induction, while 1 mM NaB significantly decreased PDLSC osteogenesis both in vitro and in vivo*.*

In addition to being an HDAC inhibitor, butyrate can bind to G-protein receptors, including GPR41/FFAR3 and GPR43/FFAR2, which are expressed in many tissue types and play various roles in physiological functions. *FFAR2*^−/−^ mice exhibited a significant increase in orthodontic tooth movement and a reduction in osteoblast counts compared to those in wild-type mice, while ALP was upregulated in the alveolar bone of *FFAR2*^−/−^ mice [21]. In human adipose-derived mesenchymal stem cells, FFAR2 upregulation was observed under conditions of adipogenic differentiation, while FFAR3 expression was below the detection limit [22]. Therefore, butyrate may exhibit converse functions during the adipogenic and osteogenic differentiation of MSCs by acting as an FFAR agonist. Here, we used an FFAR2 antagonist and agonist to confirm that NaB inhibited the osteogenic differentiation of PDLSCs by downregulating Smad1 via FFAR2 and reducing ALP activity, mineralised nodule formation, and Runx2, OCN, and OPN expression. However, in the antagonist group, ALP activity and the expression of OCN significantly reduced (compared to the control group). Thus, NaB may affect PDLSC osteogenesis by activating FFAR3.

This study demonstrates the inhibitory effect of NaB on human PDLSC viability and osteogenic differentiation capability. By activating FFAR2, NaB inhibits the expression of Smad1, thereby downregulating osteogenesis-related genes and proteins. Our findings indicated that butyrate—a major subgingival bacterial metabolite—exhibited an important inhibitory effect on PDLSC osteogenic differentiation and periodontal tissue regeneration. It has been confirmed that many signalling pathways, such as the Smad, MAPK, and NF-κB pathways, are related to MSC osteogenic differentiation [31–33]. Here, we only explored the effect of NaB on the Smad pathway. Thus, further studies are required to explore the effects of NaB on signalling crosstalk.

At present, the prevalence of periodontal disease in adults is still at a high level. The improvement of periodontal disease treatment is the key factor to improve periodontal condition. The application of MSCs to the regeneration of periodontal tissue in patients with periodontal disease is the most advanced treatment method at present. This study found that NaB inhibits the viability and inhibits the osteogenic differentiation in PDLSCs. The results suggest that NaB may affect the therapeutic effect of MSCs. Therefore, exploring the pathogenic mechanism of butyrate and finding ways to block the production and combination of butyrate are urgent problems to be solved.

## Conclusions

In summary, our results indicated that butyrate inhibits human PDLSC osteogenesis by activating FFAR2 to suppress Smad1 activation. Our findings contribute to the understanding of mechanisms underlying the following: (1) effects of butyrate on PDLSC functions and (2) effects of butyrate on periodontal tissue regeneration.

## Supplementary Information

**Additional file 1: Fig. S1.** The dose optimization assay of free fatty acid receptor 2 (FFAR2) antagonist (GLPG0974). **a** The cell proliferation curve of PDLSCs was depicted after the treatment of GLPG0974 for 24, 48, and 72 h. **b** The absorbance values showed that 20 μM GLPG0974 inhibited PDLSC proliferation at 24 h and 72 h, and 40 μM GLPG0974 inhibited PDLSC proliferation at all time points. **c** Western blotting and densitometric analysis showed that 10 μM and 20 μM GLPG0974 reversed the NaB-mediated reduction of p-Smad1/5/8 (compared to the NaB group) at 24 h. The length of the blots was appropriate in the manuscript. Error bars represent the standard deviation (n = 3). *p < 0.05, **p < 0.01, using one-way ANOVA.

**Additional file 2: Fig. S2.** The dose optimization assay of free fatty acid receptor 2 (FFAR2) agonist (4-CMTB). **a** The cell proliferation curve of PDLSCs was depicted after the treatment of 4-CMTB for 24, 48, and 72 h. **b** The absorbance values showed that 20 μM 4-CMTB inhibited PDLSC proliferation at 72 h. **c** Western blotting and densitometric analysis showed that 5, 10, 20 and 40 μM 4-CMTB decreased the expression of p-Smad1/5/8 at 24 h (compared to the control group). The length of the blots was appropriate in the manuscript. Error bars represent the standard deviation (n = 3). *p < 0.05, **p < 0.01, using one-way ANOVA.

## Data Availability

All data generated or analysed during this study are included in this published article and its supplementary information files.

## Abbreviations

SCFAs: Short chain fatty acids
MSCs: Mesenchymal stem cells
NaB: Sodium butyrate
Runx2: Runt-related transcription factor 2
OCN: Osteocalcin
Col: Type I collagen
OSX: Osterix
PDLSCs: Periodontal ligament stem cells
FFAR2: Free fatty acids receptor 2
CCK-8: Cell counting kit-8
OD: Absorbance
ALP: Alkaline phosphatase
RT-PCR: Reverse transcriptase-polymerase chain reaction
OPN: Osteopontin
HA/TCP: Hydroxyapatite/tricalcium phosphate
H&E: Hematoxylin and eosin
HDAC: Histone deacetylase
