# Supplementary figures and images for: Sodium butyrate inhibits osteogenesis in human periodontal ligament stem cells by suppressing smad1 expression
Source: BMC Oral Health. 2022 Jul 19;22:301. doi: 10.1186/s12903-022-02255-6 (PMC9297574; doi:10.1186/s12903-022-02255-6)

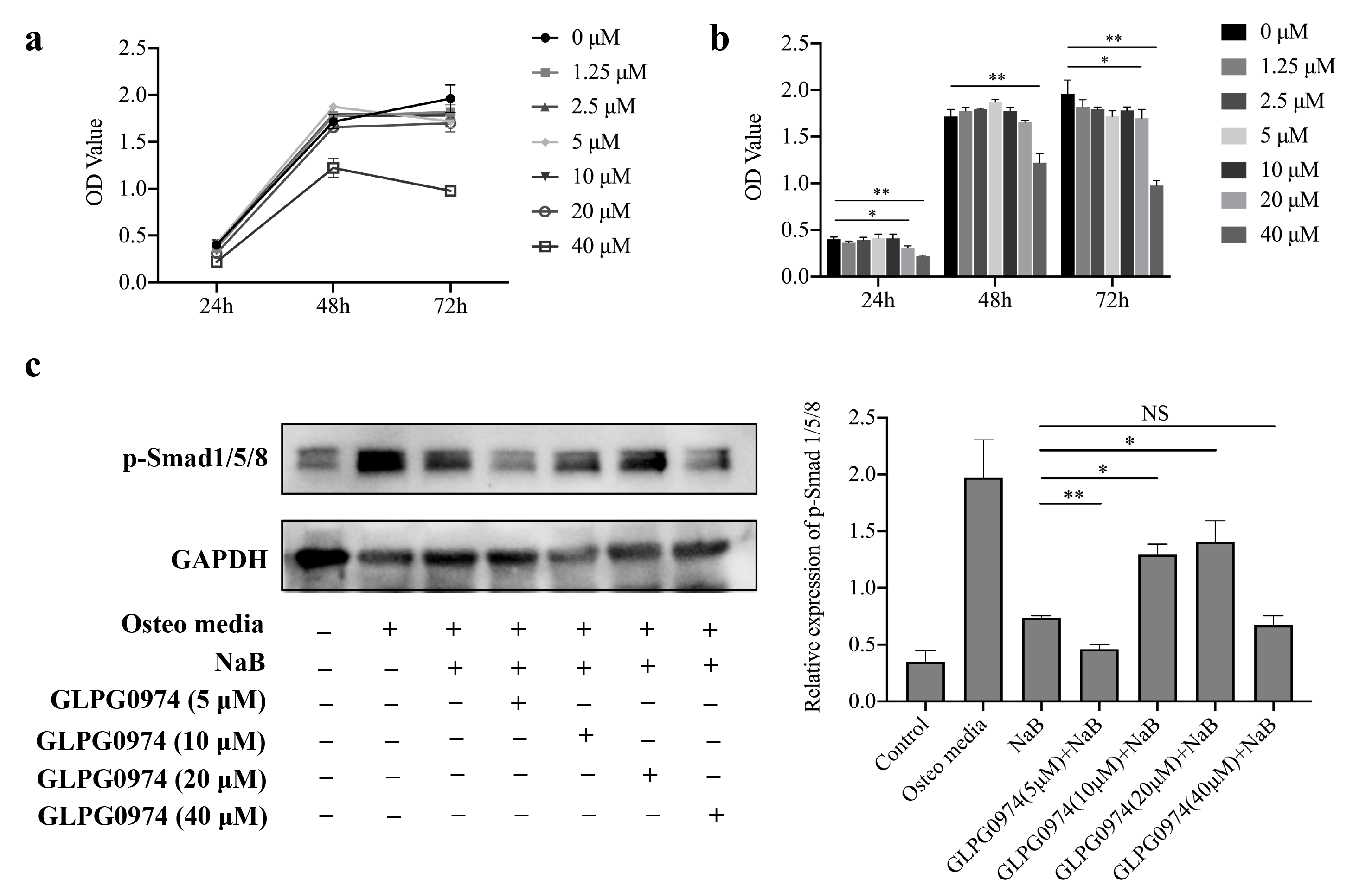

Supplement: Supplementary file 1 — Additional file 1: Fig. S1. The dose optimization assay of free fatty acid receptor 2 (FFAR2) antagonist (GLPG0974). a The cell proliferation curve of PDLSCs was depicted after the treatment of GLPG0974 for 24, 48, and 72 h. b The absorbance values showed that 20 μM GLPG0974 inhibited PDLSC proliferation at 24 h and 72 h, and 40 μM GLPG0974 inhibited PDLSC proliferation at all time points. c Western blotting and densitometric analysis showed that 10 μM and 20 μM GLPG0974 reversed the NaB-mediated reduction of p-Smad1/5/8 (compared to the NaB group) at 24 h. The length of the blots was appropriate in the manuscript. Error bars represent the standard deviation (n = 3). *p < 0.05, **p < 0.01, using one-way ANOVA. [file 12903_2022_2255_MOESM1_ESM.tif]

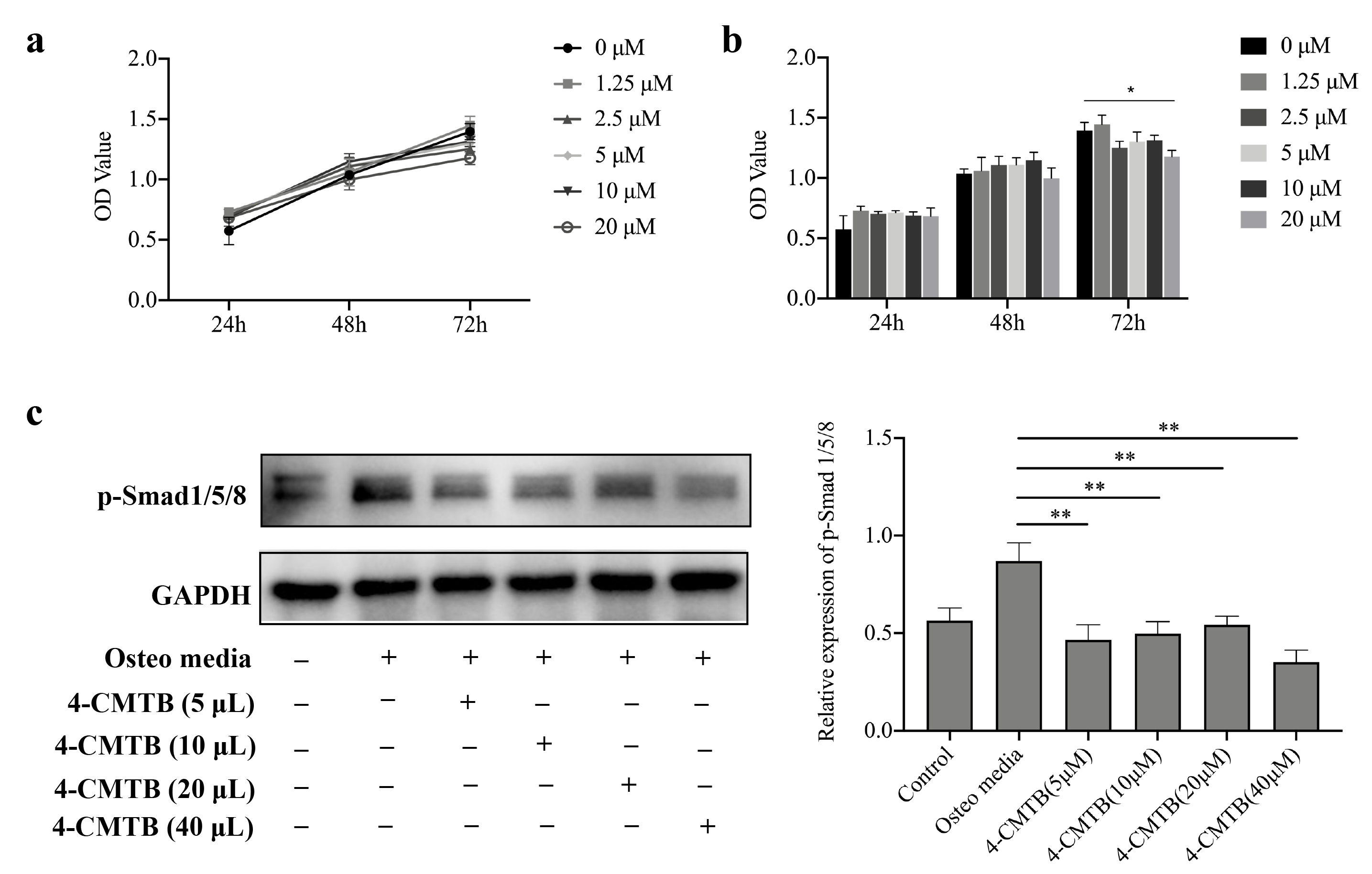

Supplement: Supplementary file 2 — Additional file 2: Fig. S2. The dose optimization assay of free fatty acid receptor 2 (FFAR2) agonist (4-CMTB). a The cell proliferation curve of PDLSCs was depicted after the treatment of 4-CMTB for 24, 48, and 72 h. b The absorbance values showed that 20 μM 4-CMTB inhibited PDLSC proliferation at 72 h. c Western blotting and densitometric analysis showed that 5, 10, 20 and 40 μM 4-CMTB decreased the expression of p-Smad1/5/8 at 24 h (compared to the control group). The length of the blots was appropriate in the manuscript. Error bars represent the standard deviation (n = 3). *p < 0.05, **p < 0.01, using one-way ANOVA. [file 12903_2022_2255_MOESM2_ESM.tif]
